# Synthesis, structure characterization, and corrosion properties of duplex electroless Ni-P/Ni-B and Ni-P/Ni-B-W coatings on mild steel

**DOI:** 10.1038/s41598-024-75883-3

**Published:** 2024-10-23

**Authors:** S. Y. Ahmed, S. B. Mahmoud, M. A. Shoeib

**Affiliations:** 1 Chemistry Department, Faculty of Women for Arts, Science and Education, Ain Shams University, Cairo, Egypt; 2 Surface Coating Department, Central Metallurgical Research and Development Institute (CMRDI), Cairo, Egypt

**Keywords:** Ni-P/Ni-B-W, Electroless deposition, Corrosion behavior, Heat treatment, Chemistry, Electrochemistry

## Abstract

This study investigates the formation of duplex electroless Ni-P/Ni-B and Ni-P/Ni-B-W alloys through electroless plating process coatings on mild steel using hypophosphite and sodium borohydride as a reducing agent, employing heat-treated. Electroless plating is affordable and suitable for coating convoluted structures. Duplex electroless on mild steel was characterized by X-ray diffraction (XRD), Energy-dispersive X-ray spectrometry (EDS), scanning electron microscopy (SEM) were used to examine the surface and cross-sectional morphologies of the duplex coating, and finally study electrochemical corrosion properties. The analysis reveals that duplex coating yields a thicker, more homogeneous coating with a characteristic cauliflower morphology and spherical nodular structures. The coating was initially amorphous, but finally crystallized when heated to 400 °C. More corrosion resistance was found in the Ni-P/Ni-B and Ni-P/Ni-B-W layers when Ni-B served as the outer covering. This study focuses on the important effects of varying tungsten concentrations and heat treatment on the corrosion resistance, surface quality, and microstructural characteristics of duplex coatings. Showed improved corrosion resistance when exposed to 0.5 g/L of Na_2_WO_4_.

## Introduction

 By chemically reducing liquid metal ions on an activated base material, a coating is created during the process known as “electroless plating,” which does not require the use of outside electricity. The plating bath contains a reducing agent, which causes this reduction. Compared to other materials used, mild steels have a high tensile strength, are less expensive, and have limited usage at high temperatures because of their poor oxidation resistance^1^. A useful method for preventing corrosion on steel and light alloy substrates is electroless nickel [EN]^2^. Because of their adaptability, some electroless nickel-based deposition techniques have seen an increase in interest among some researchers. EN plating is well known as a defensive covering in various industries because of the plausibility of plating various materials and forms with uniform coverings that display a consistent layer and excellent attachment^3^. Numerous advantages come with this approach, including low temperature, autocatalytic reactions, good selectivity, and low cost, excellent hardness, excellent lubrication, and strong resistance to corrosion and wear^4^. A reduction agent, a complexing agent, a stabilizing agent, a metal ion generator^5^, and a pH controller are frequently found in autocatalytic solutions. The electroless nickel process utilizes a variety of reducing agents, such as sodium hypophosphite^6–9^, sodium borohydride^10–13^, dimethylamine borane^14,15^, and hydrazine^16,17^. Using a reducing agent that includes boron to catalyze the reduction of nickel ions, an electroless Ni-B covering is created using materials such as sodium borohydride and dimethylamine borane. The most effective reagent for reduction is sodium borohydride^18^. A homogeneous coating with calculable amounts of nickel boride is produced by the electroless Ni-B covering procedure, which results in a substantial improvement in the wear and abrasion properties^19,20^. For the promising wear and corrosion resistance characteristics of electroless nickel^21–23^.It is feasible to use different applications on different surfaces^24^. Hypophosphite (Ni-P) baths, the most popular method of electroless nickel coating, are in some ways better than boron and hydrazine decreased baths. Ni-P baths offer higher corrosion resistance and are typically inexpensive^22,25,26^. However, the best-reducing substance for electroless nickel plating is sodium borohydride, because of its great hardness and resistance to wear. In the automotive and aerospace sectors, Ni-B coatings are especially used^27–29^. Although Ni-B coatings are not as Ni-P coatings, they have discovered numerous applications in industries^30,31^, for example, in nourishment, oil, petrochemical, chemical, plastics infusion, optics, aviation, guns, cars, and hardware. When multiple layers are combined into one coating structure, a multilayered coating. As of late, double-layer electroless coverings, such as Ni-P/Ni-B^32–35^ and Ni-P/Ni-W-P^36^, have been to enhance the ability of a conventional single Ni coating to resist corrosion. Electroless Ni-B coverings are attracting extensive interest these days because of their exceptional properties, such as cost-adequacy, thickness consistency^37^, great wear-resistance^38^, lubricity^39^, great flexibility^40^, corrosion resistance^41^, amazing solderability^42^, electrical properties, and antibacterial properties^43^. Without the use of stabilizers, the coating has a lower hardness and improved corrosion performance^44–46^. Nickel-boron-thallium and nickel-boron-lead coatings are examples of electroless nickel-boron coatings because these heavy metals are included in the coatings while stabilizers are present^47^. The highest hardness and wear resistance are found in electroless nickel-boron-lead, both in the unheated form and after heat treatment^48^. In the present investigation, two Ni-P and Ni-B monolayers, as well as two Ni-P/Ni-B and Ni-P/Ni-W-B bilayer covers, were applied using an electroless technique to mild steel. a thorough evaluation of coatings with varying Na_2_WO_4_ concentrations in terms of their structural, morphological, surface, and corrosion behavior. The obtained results show that the excellent corrosion resistance in 3.5% NaCl is worsened both as-plated and after heat treatment.

## Materials and methods

### Sample recipe

Mild steel pieces on which the coatings were made require little preparation for coating and can undergo unrestricted thermal treatment. The chemical composition comes from an iron and steel factory in Helwan. The mild steel substrates were divided into$\:\:\:24\times\:24\times\:1\:{mm}^{3\:}$segments. For easy handling, a 1 mm diameter hole was drilled near each piece’s border. To ensure a uniform surface state between the samples, using emery paper, the sample surface was smoothed to 1200 grains. The samples were mechanically prepared, cleaned with alcohol, etched for 1 min. in 30 vol% HCl, and then submerged in the coating solution. In this research, mild steel was used, and the following analyses: C 0.26%, Mn 0.6%, Si 0.08%, P 0.008%, S 0.006%, and balanced Fe were provided by the Central Metallurgical Research Institute (CMRDI).

### Electroless nickel baths

Table 1 contains a list of the constituents of Ni-P, which is the inner component of the Ni-P/Ni-B-W structure in which acidic hypophosphite was used. Each chemical component was acquired from Sigma-Aldrich. Table 2 shows the Ni-B EN plating bath constituents. The Ni^2+^ supply was nickel chloride (NiCl_2_.6H_2_O-99%), the reducing agent was sodium borohydride (NaBH_4_-99.9%), and the stabilizer was lead (II) nitrate (Pb(NO_3_)_2_-99.9%). Because of its ability to maintain activity in the bath’s alkaline pH, which was modified to (12.5-13.5) by sodium hydroxide (NaOH-98%), ethylenediamine (NH_2_-CH_2_-CH_2_-NH_2_-99%), was selected as the complexing agent. Ni-P and Ni-B layers were electrolessly plated one after the other using acidic and alkaline solutions, respectively.

**Table 1:** Electrolyte composition and plating parameters to prepare the Ni-P electroless coatings.

Chemical compounds	Concentration (g/L)	Plating parameters
Nickel sulfate	20	pH of the solution = 4.8-5
Sodium hypophosphite	15	Bath temperature = 90°C
Lactic Acid	30	
Propionic Acid	3	Plating time=60 min

**Table 2:** Electrolyte composition and plating parameters to prepare the Ni-B-W electroless coatings.

Chemical compounds	Concentration (g/L)	Plating parameters
Nickel chloride	40	pH of the solution=12.5-13.5
Sodium borohydride	1.2	Bath temperature=80°C
Ethylenediamine	90	Plating time=60 min
Sodium tungstate	0.5, 0.7, 1	
Sodium hydroxide	90	
Lead nitrate	0.0145	

### Surface characterization

SEM (JEOL-JSM-5410, Japan) with an energy dispersive spectrometer unit (EDS-Oxford) was used to investigate the plating surface morphologies and elemental analysis of the coated layer. An X-ray diffractometer (Bruker AXS-D8, Advance, Germany, controlled at 35 KV and 45 mA with CuK radiation = 0.1540 nm) was used to determine the structure of the distinct phases of the film before and after heating.

### Corrosion studies

The observations of potentiodynamic polarization were carried out in a standard three-electrode cell with a 3.5% NaCl solution using a computerized galvanostat/potentiostat (Auto LAB PGSTAT 302 N) included with the Nova program for data collection and analysis. The visible surface area of each sample was 0.385 cm^2^, and the working electrode was a mild steel sample coated with Ni-P, Ni-B, Ni-P/N-B, or Ni-P/Ni-B-W (0.5-1.0 g/L) as deposited and treated with heat. Ag/AgCl and platinum sheets were used as reference and counter electrodes. The corrosion potential (E_corr_) and corrosion current density (I_corr_) were derived from the polarization curves using the Tafel extrapolation technique, electrochemical impedance spectroscopy (EIS) observations were performed at frequencies between 100 kHz and 10 mHz. All tests were carried out in an aerated solution at a temperature of 25 °C.

## Results and discussion

### Phase composition of the covering

The following chemical equations can be used to describe electroless Ni-B coating synthesis reactions^49^:

1 $$\:2{Ni}^{2+}\:+\:{{BH}_{4}}^{1-\:}+\:{4OH}^{-}\:\to\:\:2Ni\:+\:{{BO}_{2}}^{-}\:+\:{2 H}_{2}O\:+\:{2 H}_{2\:}\:\:\:\uparrow\:\:\:\:\:\:$$

2 $$\:{{BH}_{4}}^{1-}\:+\:{2 H}_{2}O\:\to\:2B+{2OH}^{-}{+\:\:5 H}_{2}\:\uparrow$$

Nickel ions are thought to be reduced by reaction (1), and the boron content in the developed coatings is ensured by reaction (2).

**Fig. 1:**
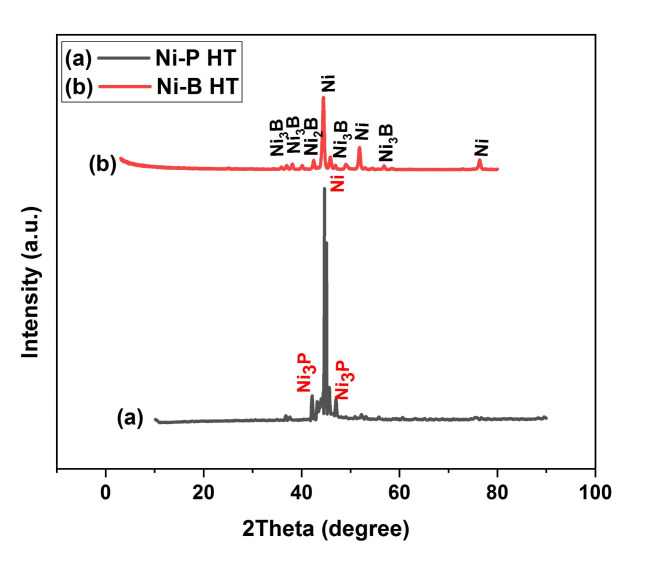
Peaks of X-ray thin film diffraction for electroless (**a**) Ni-P and (**b**) Ni-B after heating at (400 °C for 1 h).

**Fig. 2:**
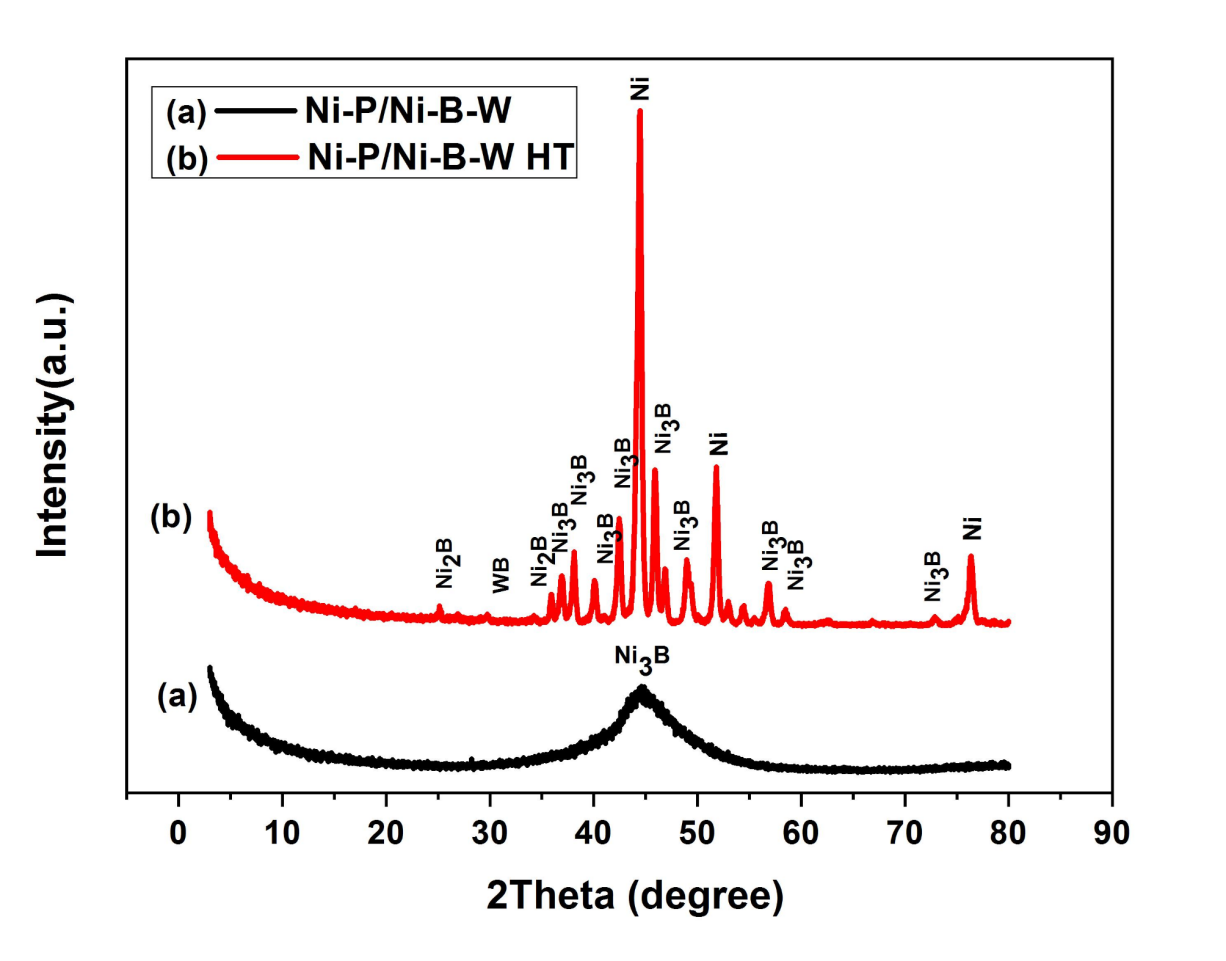
Peaks of X-ray thin film diffraction for electroless (**a**) Ni-P/Ni-B-W as plated coatings (0.5 g/L Na_2_WO_4_) (**b**) Ni-P/Ni-W-B (0.5 g/L Na_2_WO_4_) after heating at 400 °C for 1 h.

The Ni-P and Ni-B XRD patterns after a one-hour heat treatment (400 °C) are displayed in Fig. 1. These two deposits, the Ni-P deposit (Fig. 1(a)), which produces nickel and nickel phosphide (Ni_3_P), and the Ni-B deposit (Fig. 1(b)) clearly crystallize and yield nickel and nickel borides (Ni_2_B and Ni_3_B). Upon heat treatment, it is reasonable to anticipate that the duplex coating will contain Ni_3_P and Ni_3_B phases^35^. The pre-and post-heat treatment XRD thin film patterns for Ni-P/Ni-B-W are displayed in Fig. 2. (a) demonstrates that there is only one wide peak with respect to nickel visible in the Ni-P/Ni-B-W film in its as-deposited phase, making the film almost amorphous. A wide peak at approximately 2θ = 44.9 prevents the nucleation of the nickel phase and produces an amorphous structure^50,51^. Figure 2. (b) the phase transition of the Ni-P/Ni-B-W is favored by heat treatments at 400 °C for 1 h. The number of sharp peaks demonstrates that the Ni-P/Ni-B-W thin films are fully crystallized after heating and are composed of orthorhombic Ni_3_B nickel boride (#00-048-1223), tetragonal Ni_2_B (#01-082-1697), cubic Ni(#01-087-0712), and orthorhombic WB(#00-006-0541). The formation of Ni_3_B and Ni_2_B is what has caused the rise in hardness^52^.

### Surface morphology

**Fig. 3:**
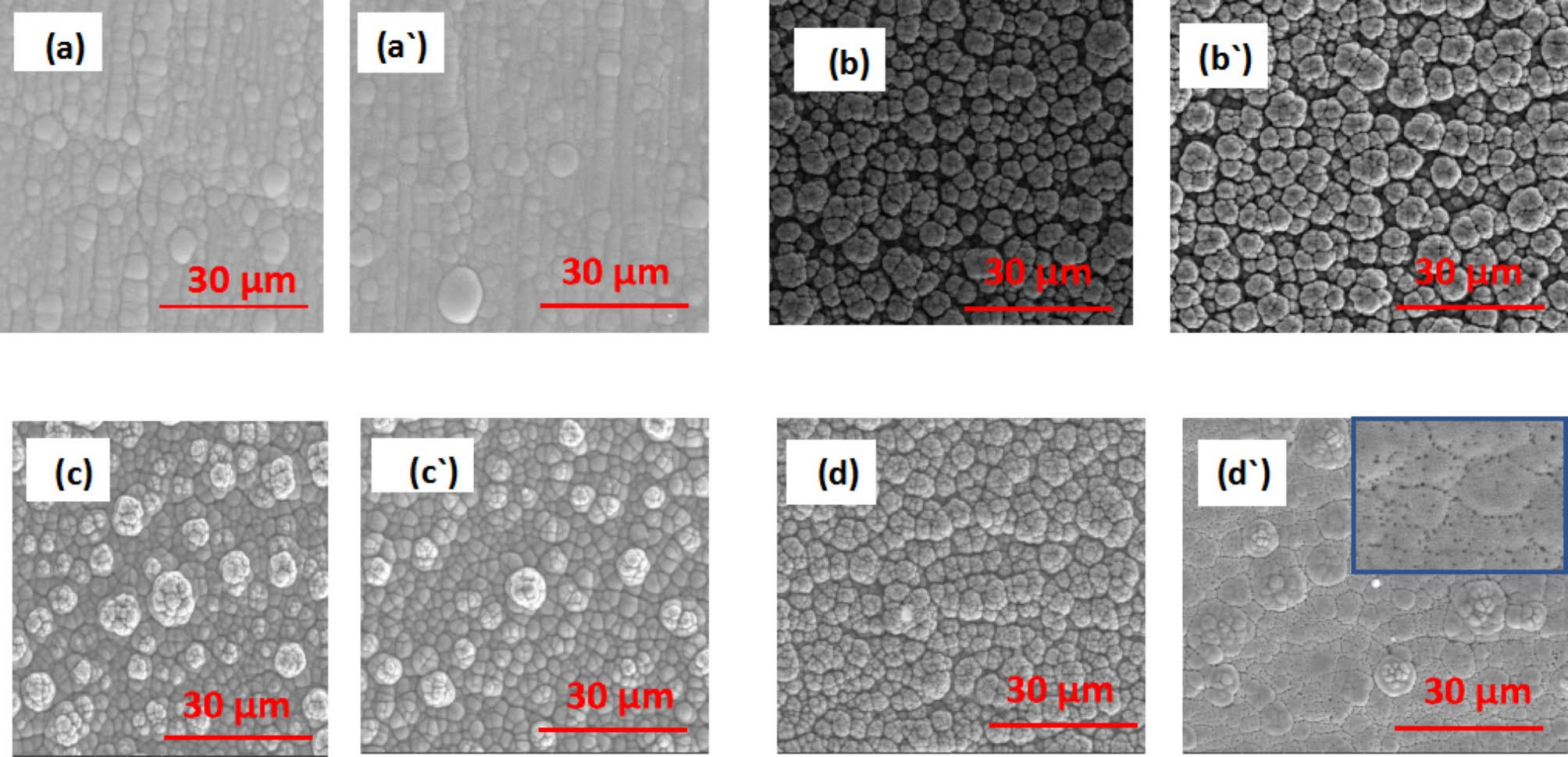
SEM micrograph before corrosion in 3.5% NaCl for (**a**) Ni-P (**a**ˋ) Ni-P HT (**b**) Ni-B (**b**ˋ) Ni-B HT (**c**) Ni-P/Ni-B (**c**ˋ) Ni-P/Ni-B HT (**d**) Ni-P/Ni-B-W (0.5 g/L Na_2_WO_4_) (**d**ˋ) Ni-P/Ni-B-W (0.5 g/L Na_2_WO_4_) HT.

Figure 3 shows the 4000×SEM images for electroless materials before corrosion in a solution of 3.5% NaCl. A single layer of electroless Ni-P is presented in Fig. 3(a) and 3(aˋ) before and after heat treatment, respectively, demonstrating the common spherical nodular structures. Figure 3(b) and 3(bˋ) demonstrate the structure of a single electroless Ni-B layer, which has a prominent characteristic cauliflower-like structure and a nodule-shaped trait. The SEM micrograph of Ni-B coatings exhibits a characteristic trait resembling a cauliflower. It is evident from the heat treatment results obtained by EDS that as nickel grain size declines and B content in Ni-B coatings rises, Ni-B deposits become harder^53^. The Ni-B coating’s lone layer has a few tiny pores that can be seen. These holes might have developed because of hydrogen evaporation while the Ni-B coating was being electrolessly deposited. Figure 3(c) and (cˋ) show the SEM images of the top layers of Ni-B and the morphology of the double layer of electroless Ni-P/Ni-B in the duplex covering. An intriguing aspect is how the Ni-P sublayer affects the surface morphology of the Ni-B top layer^54^. The Ni-B spherical nodules are more compressed (have a Ni-high P/Ni-B covering)^55^. The development of typical spherical nodular structures was discernible in Ni-P/Ni-B-W SEM micrographs obtained both before and after heat treatment (Fig. 3(d) and (dˋ)). The as-deposited (room temperature) coatings exhibited a cauliflower-like and spherical nodular structures structure. Each spherical nodule appears to be created by the fusion of numerous populations of grains that have thinned out and become prominently grained^56^. The Ni-P/Ni-B-W coating to heat treatment at 400 °C a partial transformation in surface morphology had some microcracks. The comparatively larger tungsten atoms in the coating layer may have placed internal stress on the material, which created the microcracks. The framework’s microcrack count was found in )Fig. 3(dˋ)^57,58^ .

**Fig. 4:**
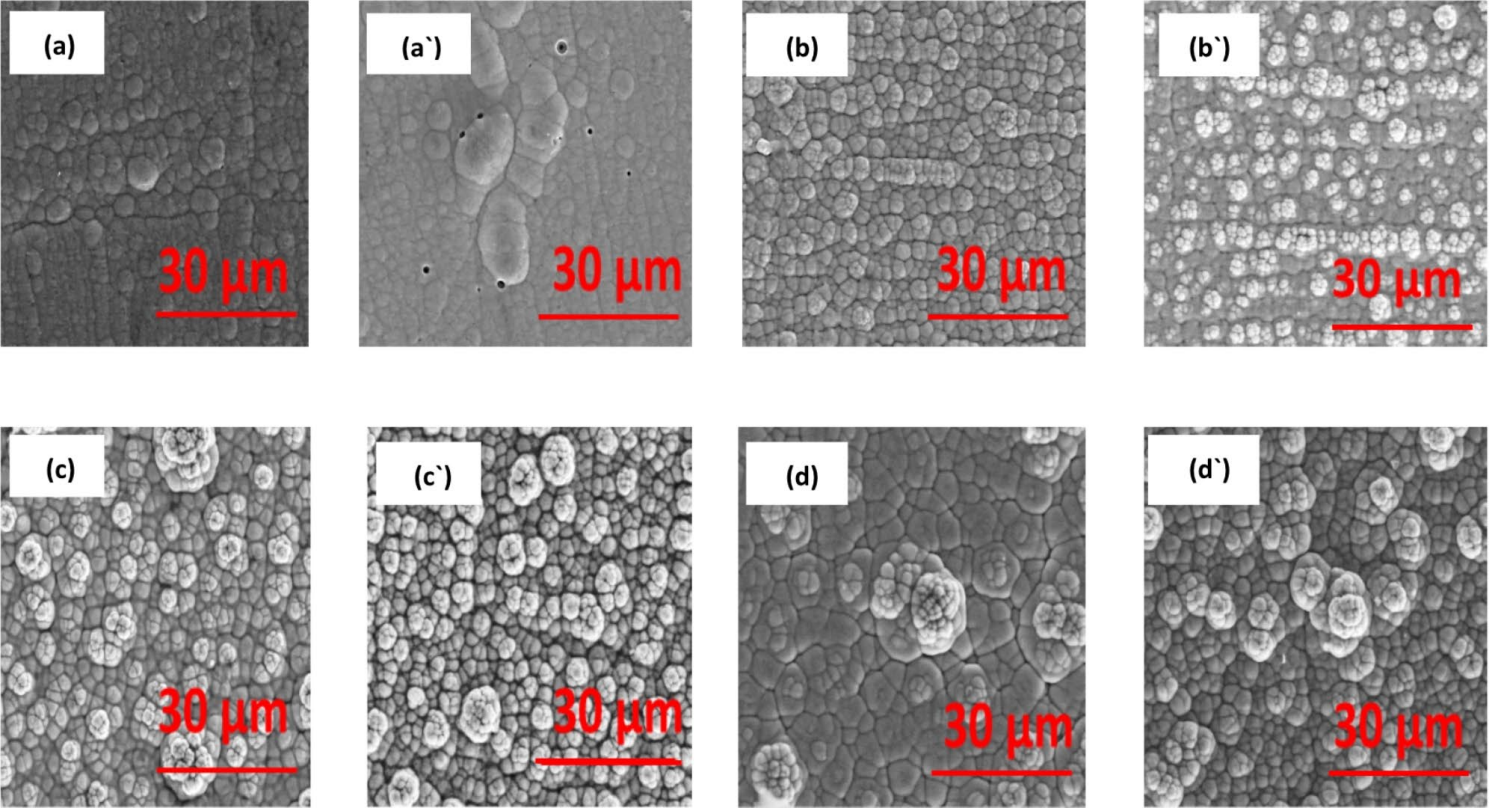
SEM micrographs after corrosion in 3.5% NaCl for (**a**) Ni-P (**a**ˋ) Ni-P HT (**b**) Ni-B (**b**ˋ) Ni-B HT (**c**) Ni-P/Ni-B (**c**ˋ) Ni-P/Ni-B HT (**d**) Ni-P/Ni-B-W (0.5 g/L Na_2_WO_4_) (**d**ˋ) Ni-P/Ni-B-W (0.5 g/L Na_2_WO_4_) HT.

After corrosion in a solution of 3.5% NaCl. A single layer of electroless Ni-P is presented in Fig. 4 (a) and Fig. 4(aˋ) before and after heat treatment, respectively, showing the typical spherical nodular structures. However, in Fig. 4(aˋ) llustrates surface with obvious defects were present on the surface after heat treatment due to a higher P content in the covering following heat treatment than an unaltered coating (in Table 4)^59^.

Figure 4(b), (bˋ), (c), (cˋ), (d) and (dˋ) The surface morphologies are in the shape of a cauliflower structure, that the coating’s nodular structure was refined by heat treatment. After heat treatment, the coating appears uniform and crystalline. This is explicable by the fact that nickel borides created after annealing reduce the coating’s porous structure and create a denser covering^60,61^.

### Cross-section observation

**Fig. 5:**
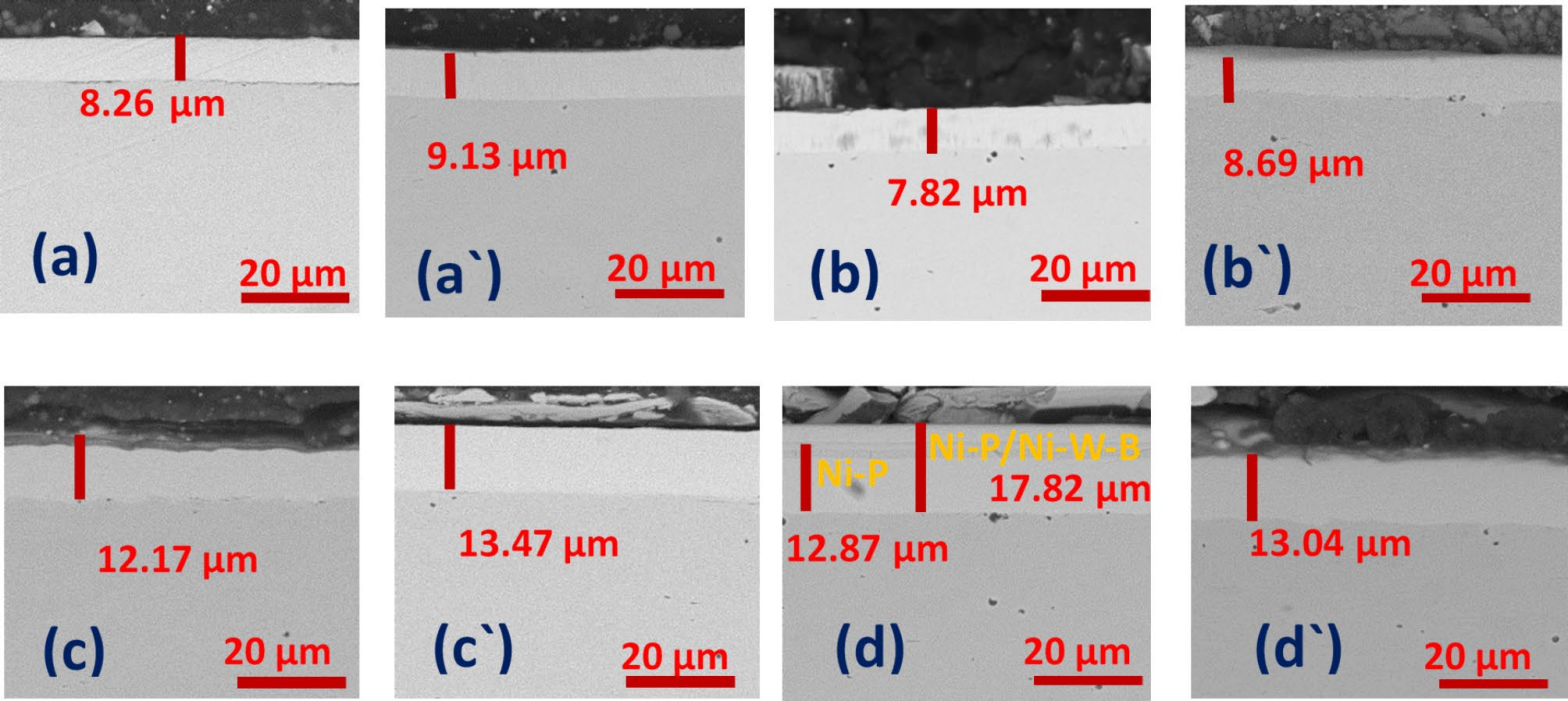
Cross portion of (**a**) Ni-P (**a**ˋ) Ni-P HT (**b**) Ni-B (**b**ˋ) Ni-B HT (**c**) Ni-P/Ni-B (**c**ˋ) Ni-P/Ni-B HT (**d**) Ni-P/Ni-B-W (0.5 g/L Na_2_WO_4_) (**d**ˋ) Ni-P/Ni-B-W (0.5 g/L Na_2_WO_4_) HT.

 Figure 5(a) demonstrates the solitary Ni-P coating’s cross-sectional microstructure. The coating can be seen to be a uniform layer with a mean thickness. The coating-substrate thickness was found to be about 8.26 μm. After heat treatment Fig. 5(aˋ) the thickness increased due to the oxidation process ^62^. Figure 5(b) shows the thickness of the Ni-B single layer, which was found to be 7.82 μm. Figure 5(bˋ) the oxidation process after heat treatment increased the thickness, which was found to be 8.69 μm. Figure 5(c) and Fig. 5(cˋ) display the Ni-P/Ni-B cross section before and after the heat process, which was found to be 12.17 μm and 13.47 μm which the oxidation process after heat treatment increased the thickness Fig. 5(d) illustrative example of the Ni-P/Ni-B-W coating. The layers have a combined thickness of roughly 17.82 μm and it has an outer Ni-B-W coating that is 4.95 μm thick and an interior Ni-P coating that is 12.87 μm thick. The duplex coating has a dense structure, and the layers and substrate successfully form a solid connection. In addition, this coating is flawlessly made and has no macro-defects. In reality, there are no voids or gaps between the layers, and they precisely follow the interface profile. A distinction between the outer and interior coatings was not observed after heat treatment (Fig. 5 (dˋ)). These findings demonstrated the Ni-P/Ni-B coating outstanding adhesion. Ni-P/ Ni-B-W HT coating cross-sections reveal a decreasing coating thickness. The manner in which the number of cracks increases is shown in the SEM figure. The enormous internal tension caused cracks at the cross-Sect. ^64^. The boron content decreases as tungsten is added to electroless baths used to manufacture Ni-P/ Ni-B-W films^64^.

**Table 3:** Chemical composition of blank, Ni-P, Ni-B Ni-P/Ni-B and Ni-P/ Ni-B-W (0.5 g/L Na_2_WO_4_) prior to and following thermal treatment, as determined by EDS results.

	Ni (Wt.%)	Fe (Wt.%)	*P* (Wt.%)	B (Wt.%)	W (Wt.%)
blank	----	31.49	----	----	----
Ni-P	71.02	8.79	20.19	----	----
Ni-P HT	64.97	8.67	26.36	----	----
Ni-P/Ni-B	66.39	8.68	12.44	12.49	----
Ni-P/Ni-B HT	39.78	5.46	14.13	40.63	----
Ni-P/ Ni-B-W	75.31	7.20	10.54	03.49	3.46
Ni-P/ Ni-B-W HT	69.05	7.02	11.06	09.13	3.74

The chemical compositions of the mild steel (blank), Ni-P, Ni-P HT, Ni-P/Ni-B, Ni-P/Ni-B HT, Ni-P/ Ni-B-W and Ni-P/ Ni-B-W HT deposits are given in Table 3. High phosphorus coatings have (10.54-26.36 Wt.% ) offers the excellent corrosion protection. Tungsten added to the coating as a third particle in a duplex coating is decreases the B and P content, which is expected to have positive effect on behavior of the coating, where the B content in Ni-P/Ni-B was 12.49 Wt.% decease to 10.54 Wt.% in Ni-P/ Ni-B-W. Since there is less boron and phosphorus in Ni-P/Ni-B and Ni-P/Ni-B-W than there is in heat treatment, the coating offers higher corrosion protection for the material as it is deposited^65^.

### Corrosion tests

**Fig. 6:**
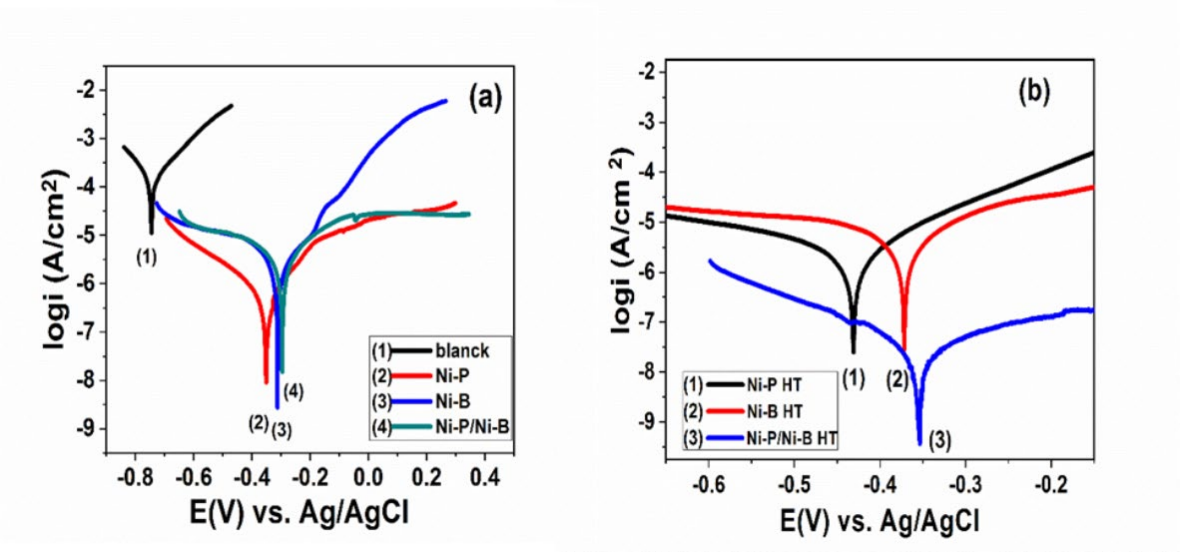
(**a**,**b**) Various polarization curves of blank, Ni-P, Ni-B and Ni-P/Ni-B with and without heat treatment exposure to 3.5% NaCl solution at room temperature.

**Fig. 7:**
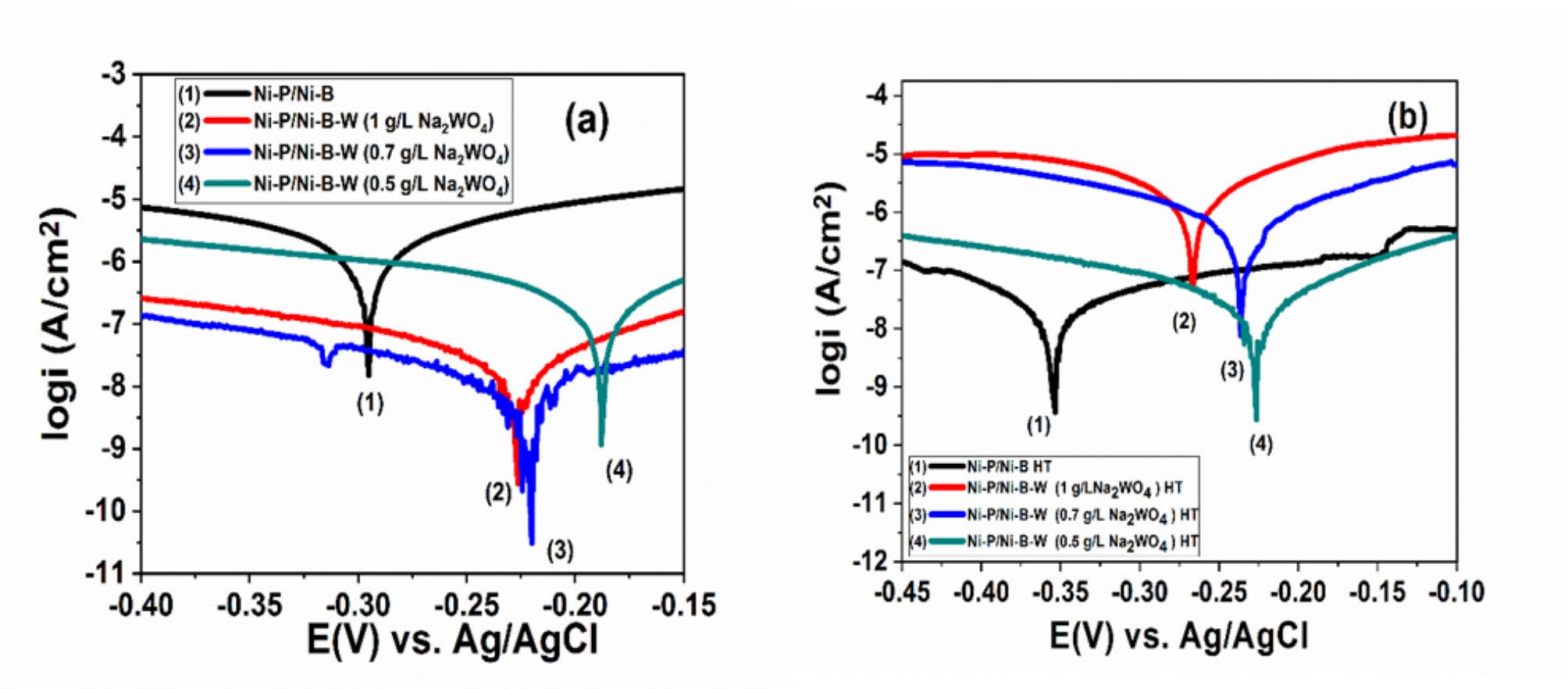
(**a**,**b**) Polarization curve of different coatings Ni-P/Ni-B and Ni-P/Ni-B-W with and without heat treatment immersion at 400 °C in a 3.5% NaCl solution.

**Table 4:** Polarization curve values for electrochemical characteristics of blanck, Ni-B, Ni-P and douple Ni-P/ Ni-B with and without heat treatment.

Sample	E _corr_ (mV)	I _corr_ (µ A/cm^2^)	*R*_i_ (mm/y)	*R*_*p*_ (KΩ.cm^2^)
blank	-0.748	7.200	0.0823	9.5400
Ni-P	-0.372	4.022	0.0420	7.2546
Ni-B	-0.354	3.620	0.0228	8.2378
Ni-P/ Ni-B	-0.298	2.180	0.0039	11.023
Ni-P HT	-0.434	4.930	0.0991	4.9100
Ni-B HT	-0.374	4.150	0.0394	7.3158
Ni-P/Ni-B HT	-0.362	3.920	0.0320	3.4502

Corrosion protection performance of mild steel coated with monolayer Ni-P, Ni-B, or duplex Ni-P/ Ni-B and Ni-P/ Ni-B-W coating with different tungsten concentrations (0.5-1.0 g/L Na_2_WO_4_) was investigated in 3.5% NaCl solution in Figs. 6 and 7 with and without heat treatment. The coatings were plated and heat-treated and then immersion in 3.5 wt% NaCl solution. Table 4 shows the corrosion potential (E_corr_) and current density (i_corr_) determined using the Tafel extrapolation method for Ni-P, Ni-B, and substrate, and Ni-P/Ni-B coatings with and without heat treatment. Figure 6(a) Presents the potentiodynamic polarization curves of the as-deposited coatings and is compared with the mild steel substrate. In the current investigation, when compared to Ni-P, Ni-B, and substrate, and Ni-P/Ni-B coatings. Electroless nickel coatings reduced with borohydride have better corrosion resistance compared to those reduced with sodium hypophosphite. The coatings samples show better behavior: there is a decrease in corrosion current densities (i_corr_) while a positive shift in corrosion potential (E_corr_) occurs. While the corrosion potential of mild steel is -0.748 V, the potential values of Ni-P and Ni-B coatings samples increase to -0.372 and − 0.354 mV, respectively. While the corrosion current density of mild steel is 7.200 µ A/cm^2^, the icorr values of Ni-P and Ni-B coatings samples decrease to 4.022 µ A/cm^2^ and 3.620 µ A/cm^2^, respectively. Duplex coating Ni-P/Ni-B appear nobler and have a slower corrosion rate and a lower corrosion current density^66^. Figure 6(b) demonstrates the electrochemical polarization profiles of monolayer Ni-P, Ni-B coatings, and duplex Ni-P/Ni-B coatings at heat treatment in 3.5 wt% NaCl solution. All coatings exhibited significant increases in corrosion potential and a clear reduction in corrosion current density. Figure 7 displays the electrochemical polarization graphs of as-plated, heat-treated Ni-P/Ni-B, and Ni-P/Ni-B-W coatings at various Na_2_WO_4_ (0.5-1.0 g/L) concentrations in a 3.5% NaCl aqueous medium at room temperature (roughly 25 °C). Table 5 provides an overview of the corrosion potential and corrosion current intensity. The most promising material is duplex Ni-P/Ni-B and Ni-P/Ni-B-W(0.5-1.0 g/L), which is also thought to have greater corrosion protection than a monolayer. In addition, as the tungsten concentration employed to produce the films declined, the corrosion properties improved. Comparable-thickness Ni-P and Ni-B covers are less corrosion-resistant than Ni-P/Ni-B and Ni-P/Ni-B-W (0.5 g/L Na_2_WO_4_) layers. The coating as deposited Ni-P/Ni-B-W (0.5 g/L) have lowest current density 0.3856 µA/cm^2^ and corrosion potential -0.188 mV represents the best corrosion behavior compared with others. Tables 4 and 5 show how heat treatment affects corrosion current density (I_corr_). It is common knowledge that the addition of tungsten to an electroless nickel-boron coating system usually improves corrosion resistance. The layer’s heat treatment reduced corrosion resistance due to the presence of cracks after heat treatment^67,68^. The main cause for the difference in corrosion resistance between as-plated and heat-treated coatings exposed to chloride ions was clearly the transition of the microstructure from an amorphous to a crystalline shape. Tungsten tends to produce an oxide film on the surface, which boosts corrosion resistance when added to an electroless nickel-boron coating system^69^. The corrosion resitance of both mono or duplex coating before heat treatment is higher than that after heat treatment as shown in Tables 4 and 5.

**Table 5:** Polarization curve values for electrochemical characteristics of as-plated, heat-treated Ni-P/Ni-B-W coatings at various Na_2_WO_4_ concentrations in a 3.5% NaCl aqueous medium at room temperature.

Sample	E _corr_ (mV)	I _corr_ (µA/cm^2^)	*R*_i_(mm/y )	Rp (KΩ.cm^2^)
Ni-P/ Ni-B-W (0.5 g/L Na_2_WO_4_)	-0.188	0.3856	0.00054	15.4687
Ni-P/ Ni-B-W (0.7 g/L Na_2_WO_4_)	-0.221	0.9587	0.00085	14.5812
Ni-P/ Ni-B-W (1 g/L Na_2_WO_4_)	-0.231	1.3184	0.00096	13.9874
Ni-P/ Ni-B-W (0.5 g/L Na_2_WO_4_) HT	-0.226	1.3505	0.00120	13.5840
Ni-P/ Ni-B-W (0.7 g/L Na_2_WO_4_) HT	-0.236	1.9120	0.00190	12.8940
Ni-P/ Ni-B-W (1 g/L Na_2_WO_4_) HT	-0.262	2.0582	0.00240	12.1427

**Fig. 8:**
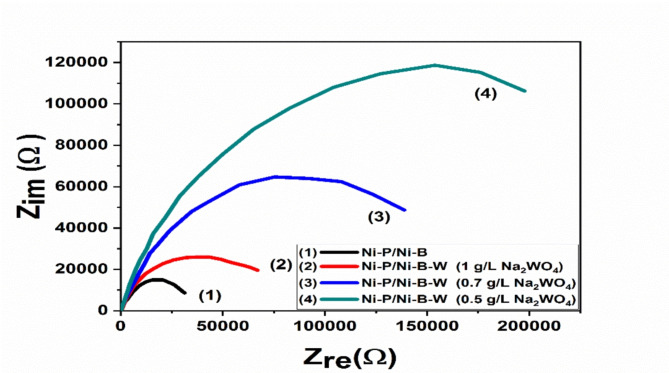
Electroless Nyquist plot curves for Ni-P/Ni-B and Ni-P/Ni-B-W (0.5 g/L Na_2_WO_4_) coatings were acquired before the coatings were heated in 3.5% NaCl.

**Fig. 9:**
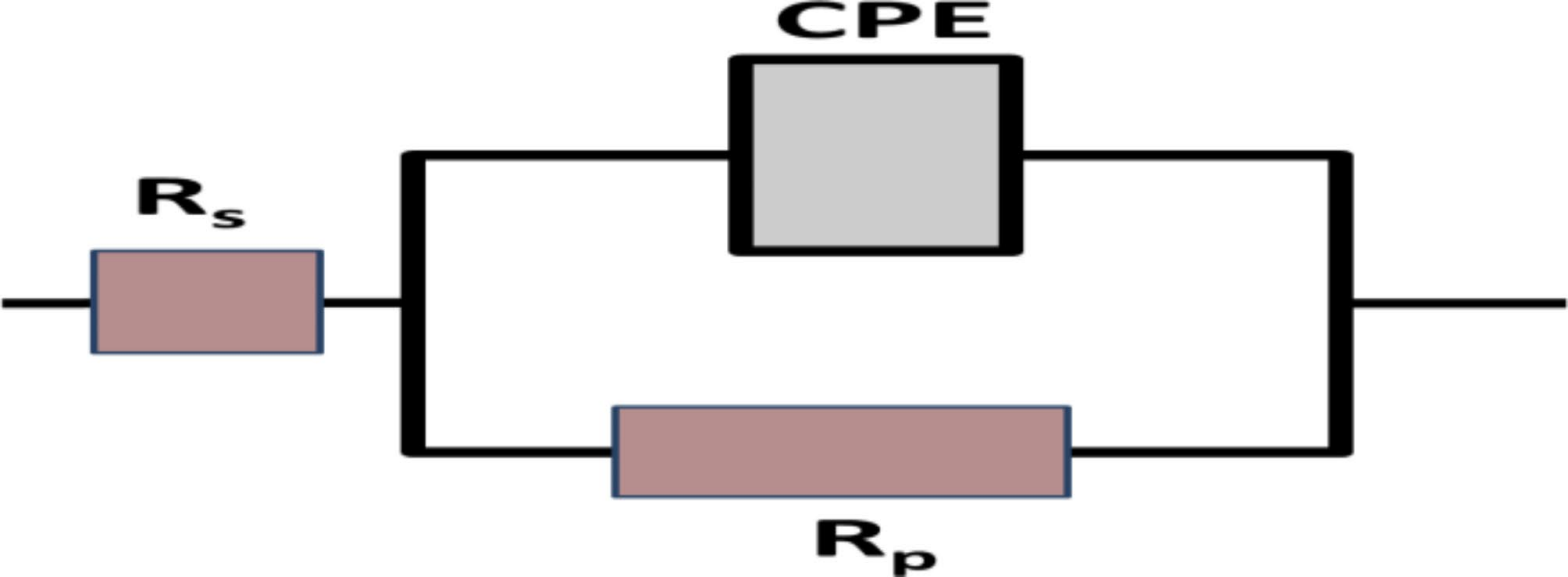
Equivalent circuits used in the investigation of EIS modeling.

The Nyquist plot of a mild steel alloy coated with a Ni-P/Ni-B or Ni-P/Ni-B-W coating with varying tungsten concentrations (0.5-1.0 g/L Na_2_WO_4_) was examined using electrochemical impedance spectroscopy (EIS), and the results are shown in Fig. 8. The impedance study supports the polarization results obtained earlier. The Ni-P/Ni-B-W coverings exhibit the greatest corrosion performance, and the corrosion resistance increases in the layer with tungstate concentrations of 0.5 g/L Na_2_WO_4_. One capacitive semicircular loop formed the obtained Nyquist graphs. In every instance, a decline in tungstate concentration results in a growth in the semicircle size, which denotes an increase in coating resistance. The simplest model with a one-time constant, as shown in Fig. 9, is the electrical model that best captures the rusting process. A constant phase element (CPE) that is parallel to the coating surface Rp’s polarization resistance (charge transfer resistance) and both connected in series with the solution resistance Rs make up the analogous circuit. Due to the surface heterogeneity of the coating, the circuit’s constant phase element (CPE), which is determined by the impedance value, compensates for the effect of the capacitance.

3 $$\rm Z_{CPE}= [C (j\omega)^\alpha]^{-1}$$

where j is an imaginary integer (j = (-1)^1/2^), ω = 2f is the angular frequency in rad/s, and f is the frequency in Hz, α is an exponent that accounts for surface heterogeneity.

## Conclusion

The following finding is reached because of the investigation findings:

1. On a mild steel substrate, high-quality and uniform electroless Ni-P/Ni-B and Ni-P/Ni-B-W layers were mostly produced, and these layers are more corrosion resistant than equivalent Ni-P and Ni-B covers.
2. Duplex coatings were found to have decreased corrosion protection after heat treatment as a result of the film’s loss of its amorphous properties, and the increase in hardness was due to the creation of Ni_3_B and Ni_2_B.
3. The duplex coatings obtained had the characteristic cauliflower structure
4. It was observed that by lowering the tungsten content in the Ni-P/ Ni-B-W (0.5 g/L) double layer coating’s cross-section were thicker and homogeneous, the layers have a dense structure and a strong bond with the substrate because of their estimated total thickness of 17.82 μm.
5. The co-deposition of W into Ni-P/ Ni-B-W reduces the phosphorus and boron concentration
6. After heating, it is evident that the nickel grain size shrinks and Ni-B deposits harden as the boron concentration in the coatings increases.
7. The effects of the addition of Na_2_WO_4_ and heat treatment on the corrosion resistance, surface quality, and microstructural characteristics of duplex coatings were studied.
8. Both duplex coatings Ni-P/Ni-B and Ni-P/Ni-B-W showed higher corrosion resistance compared to monolayer Ni-P and Ni-B and mild steel. The best result in corrosion analyses were obtained with the 0.3856 µA/cm^2^ i_corr_ value on the sample coated with Ni-P/Ni-B-W before heat treatment.
9. Adding 0.5 g/L Na_2_WO_4_ to Ni-B coating provides low corrosion tendency and rate. The wider the impedance radius, the stronger the polarization resistance, providing the best protection from corrosion.

## Data Availability

The datasets used and analysed during the current study available from the corresponding author on reasonable request.
